# The Gut Microbiota of Obese Children Releases Lower Antioxidant Capacity from Food than That of Lean Children

**DOI:** 10.3390/nu14142829

**Published:** 2022-07-09

**Authors:** Beatriz Navajas-Porras, Sergio Pérez-Burillo, Daniel Hinojosa-Nogueira, Konstantinos Douros, Silvia Pastoriza, José Ángel Rufián-Henares

**Affiliations:** 1 Departamento de Nutrición y Bromatología, Instituto de Nutrición y Tecnología de Alimentos, Centro de Investigación Biomédica, Universidad de Granada, 18071 Granada, Spain; beatriznavajas@ugr.es (B.N.-P.); spburillo@ugr.es (S.P.-B.); dhinojosa@ugr.es (D.H.-N.); spdelacueva@ugr.es (S.P.); 2 Pediatric Allergy and Respiratory Unit, 3rd Department of Pediatrics, “Attikon” University Hospital, National and Kapodistrian University of Athens, 12462 Athens, Greece; costasdouros@gmail.com; 3 Instituto de Investigación Biosanitaria ibs. GRANADA, Universidad de Granada, 18071 Granada, Spain

**Keywords:** antioxidant capacity, in vitro digestion, in vitro fermentation, obesity, children, gut microbiota

## Abstract

The prevalence of obesity has been increasing in children over the last few decades, becoming a concern for health professionals and governments. Gut microbial community structure in obese people have been found to differ from that of lean subjects for some taxa which could result in different production of microbial metabolites. The aim of the present work was to study whether the gut microbiota from obese children extracts a different concentration of antioxidant capacity than the gut microbiota from lean children. For this purpose, different foods were in vitro digested and in vitro fermented using fecal material from obese and lean children. FRAP, DPPH and Folin-Ciocalteu methods were used to measure the antioxidant capacity released during digestion and fermentation. Overall, when using lean gut microbiota, antioxidant capacity released was higher when measured via DPPH and FRAP. Moreover, according to DPPH results, lean gut microbiota could potentially release more antioxidant power from vegetables than from animal products, while obese gut microbiota did the opposite. On the contrary, with the FRAP method obese gut microbiota released higher levels of antioxidant power from plant products than from animal products, but the final antioxidant capacity was still lower than that released by lean gut microbiota. Therefore, these results reflect that the total antioxidant capacity of foods is influenced by the gut microbiota, although whether that antioxidant capacity is released from plant or animal products can be slightly influenced by the method used for analysis.

## 1. Introduction

According to the World Health Organization, obesity is defined as a preventable risk factor that involves an abnormal or excessive accumulation of fat that can be harmful to health and is one of the most serious health problems of the 21st century. In particular, the global prevalence of childhood obesity has markedly increased in recent decades and is considered a global pandemic [1]. It is estimated that 12-36% of European children aged 7–11 years are overweight or obese [2]. In addition to a number of health problems derived from obesity such as cardiovascular disease or diabetes, it is increasingly recognized that gut microbial community structure and functionality change in the context of non-communicable diseases such as obesity [1,3].

The gut microbiota is considered a metabolic organ consisting of a set of microorganisms and their genomes [1]. It is composed of members from different phyla though *Firmicutes* and *Bacteroides* are the most dominant. Obese patients have been found to show some disturbances in the community such as lower alpha diversity [4,5]. Additionally, obese patients usually show an increased *Firmicutes/Bacteroides* ratio [6,7,8,9,10,11]. There are also some bacteria that is more often found in higher abundance in lean subjects: the family Chistensenellaceae and the genera *Methanobacterial*, *Lactobacillus*, *Bifidobacteria*, and *Akkermansia* [11].

The gut microbiota plays an important role in human metabolism. In fact, it is involved in modulating host nutrition, resulting in vitamin production, fermentation of indigestible dietary components, production of short-chain fatty acids, vitamins and essential amino acids, enrichment of specific lipopolysaccharides, regulation of energy absorption, central appetite, fat storage, chronic inflammation and circadian rhythms [12,13,14]. Some of the most antioxidant compounds of the diet, phenolics, are actually not absorbed in the small intestine, reaching the colon where they are, in great extent, broken down by gut microbes [15,16]. Therefore, gut microbes can potentially have a great influence on the antioxidant capacity that we actually obtain from food. Accordingly, any disturbance in the microbial community could result in an alteration in the concentration of antioxidant capacity extracted from foods.

Different foods of animal origin (e.g., meat, fish, eggs) and most vegetables are consumed after being cooked under different conditions [17]. Heat treatments directly impact the final composition of foods [18,19] due to chemicals modifications such as the Maillard reaction [20]. In this sense, boiling is the less aggressive cooking technique compared to frying, roasting or grilling [21]. Therefore, assessing the effect of mild thermal treatments on the antioxidant capacity is also necessary. 

The aim of this work was to study the antioxidant capacity of Mediterranean diet foods after in vitro digestion and in vitro fermentation. In vitro fermentation was performed with gut microbiota obtained from fecal material from obese and lean children.

## 2. Materials and Methods 

### 2.1. Chemicals

#### 2.1.1. In Vitro Digestion and Fermentation

Pancreatin from porcine pancreas was acquired from Alpha Aesar (Kandel, Germany). Tryptone, cysteine, resazurin, sodium dihydrogen phosphate, sodium sulfide, porcine bile acids, pepsin and salivary alpha-amylase were purchased from Sigma-Aldrich (Darmstadt, Germany).

#### 2.1.2. Antioxidant Capacity

Trolox ((±)-6-Hydroxy-2,5,7,8-tetramethylchromane-2-carboxylic acid), DPPH (2,2 diphenyl-1-1picrythydrazul hydrate 95%), sodium acetate, TPTZ (2,4,6-Tri(2-pyridyl)-s-triazine), iron (III) chloride hexahydrate, gallic acid, Folin-Ciocalteu® reagent and hydrochloric acid. All the reagents for the analysis of antioxidant capacity were obtained from Sigma Aldrich (Darmastad, Germany).

### 2.2. Samples 

A total of 47 foods belonging to the following groups were studied: nuts (nut mix and peanuts), cereals (bread, whole grain bread, pasta, whole grain pasta, rice, whole grain rice, biscuits, whole grain biscuits, breakfast cereals and whole grain breakfast cereals), vegetables (zucchini, pepper, carrot, eggplant, onion, cauliflower, spinach, tomato, cabbage and lettuce), legumes (kidney beans and lentils), fruits (apple, banana, orange, grapes, plum, peach and olives), tubers (potato and sweet potato), cocoa (dark chocolate and cocoa butter), dairy (butter, gouda, milk and yogurt), meat (chicken, beef, lamb and pork) fish (salmon and cod fish) and egg. Samples were analyzed either in their raw form or submitted to boiling in water for those foods that are usually cooked. Boiling was carried out in ultra-pure water for 20 min at a 5:1 ratio (water:food) on the following foods: pasta, whole grain pasta, rice, whole grain rice, kidney beans, lentils, potato, sweet potato, chicken, beef, lamb, pork, salmon, cod fish, egg, carrot, cauliflower, onion, pepper, spinach and zucchini. The food was acquired from 3 retail shops and stored refrigerated or in a cold room, according to the label or retailer instructions.

### 2.3. In Vitro Digestion and Fermentation

Every single sample was in vitro digested and fermented following previous protocols [22,23]. For each sample, 5 g of the sample was weighed (in triplicate) for further in vitro gastrointestinal digestion and in vitro fermentation. In vitro digestion consists of 3 steps: oral, gastric and intestinal. First, samples were weighed into a 50 mL tube. A total of 5 mL of simulated salivary fluid with 150 U/mL were added and mixed into the 50 mL tube carrying the sample and kept at 37 °C for 2 min. Secondly, 10 mL of simulated gastric fluid with 4000 U/mL of gastric pepsin were added to the mix, the pH lowered to 3 and kept at 37 °C for 2 h. Finally, 20 mL of simulated intestinal fluid with 200 U/mL of pancreatin and 20 mM bile salts were added into the tube, the pH increased to 7 and kept at 37 °C for 2 h. Enzyme activity was halted by immersion in ice for 15 min. Tubes were centrifuged, the supernatant (fraction available for absorption at the small intestine) collected and the pellet (fraction not digested that would reach the colon) used for in vitro fermentation. 

Fecal samples from 5 lean donors and 5 from obese were used for the in vitro fermentation. The common inclusion criteria for both groups of children were age between 8 and 10 years as well as not having taken antibiotics or probiotics 3 months before the start of the study. Common exclusion criteria were diagnosis of chronic gastrointestinal disorders or any other chronic disease or special diet. Only lean children with a BMI between the 5th and 85th percentile for sex, height and age were considered. For the obese group, BMI had to be greater than the 95th percentile for sex, weight and age. Recruitment of the study participants was done via the pediatric unit at the hospital in Athens (Greece). Parents were given an informed consent from as well as information and questionnaires for inclusion/exclusion criteria. The study was approved by ethics committee at the University General Hospital in Athens. 

Fecal material was pooled by donor group (lean children and obese children) to account for inter-individual variability. In vitro fermentation was carried out at 37 °C for 20 h, in oscillation. For this purpose, 0.5 g of the pellet obtained after in vitro gastrointestinal digestion were used, as well as 10% of the supernatant. Fermentation medium composed of peptone (14 g/L, cysteine 312 mg/L, hydrogen sulfide 312 mg/L and resazurin 0.1% *v*/*v*) was added to the fermentation tube at a volume of 7.5 mL. A fecal inoculum mas made from fecal material by mixing it with PBS at a concentration of 33%. Two milliliters of inoculum were added to the fermentation tube. Afterwards, nitrogen was bubbled into the tube until reaching anaerobic conditions (transparent solution as opposed to pink when oxygen is dissolved). After 20 h at 37 °C, microbial activity was halted by immersion in ice for 15 min and tubes were centrifuged to collect the supernatant (fraction available for absorption at the large intestine), which was stored at −80 °C until further analysis. Blanks carrying water instead of the sample were included in the in vitro digestion as well as in the in vitro fermentation. 

### 2.4. Antioxidant Assays

Antioxidant capacity was studied in both the fraction obtained from in vitro digestion (highly in the small intestine) as well as in the fraction obtained after in vitro fermentation (absorbable in the large intestine). The sum of these two fractions would make for the total antioxidant capacity of the sample [24]. Three different methods were used to measure antioxidant capacity since different antioxidant methodologies are usually carried out under different physicochemical conditions such as pH or solvent. In addition, different antioxidant assays can reflect different redox principles or use different chemicals as donors/acceptors of electrons.

The Folin–Ciocalteu (FC) assay was followed with a previous protocol [25]. It was performed with a microplate reader (FLUOStar Omega, BMG Labtech, Ortenberg, Germany). A total of 30 μL of the samples were added in duplicate to each of the 96 wells of a plastic plate. This was mixed with 190 μL of bidistilled water, 15 μL of Folin–Ciocalteu reagent and 60 μL of 10% sodium carbonate solution. The calibration curve was prepared with gallic acid with a concentration that ranged from 0.1 to 2.5 mg/mL. The antioxidant reaction was monitored for 60 min at 37 °C. The results were expressed as mg gallic acid equivalent/kg of food. 

TEAC_FRAP_ assay (Trolox equivalent antioxidant capacity referred to reducing capacity). The protocol of Benzie and Strain [26] was followed to study the reducing capacity of iron of the different samples. The procedure was carried out in a microplate reader (FLUOStar Omega, BMG Labtech, Ortenberg, Germany). In this case, 20 μL of sample were placed in duplicate in the 96-well microplate and mixed with 280 μL of freshly prepared FRAP reagent (25 mL of 0.3 mM sodium acetate pH 3.6, 2.5 mL of 20 mM ferric chloride and 2.5 mL of 40 mM TPTZ). The antioxidant reaction was monitored for 30 min at 37 °C and the calibration curve ranged from 0.01–0.4 mg of Trolox/mL. Results were expressed as mmol Trolox equivalent/kg food.

TEAC_DPPH_ assay (Trolox equivalent antioxidant capacity versus DPPH radicals). This method was carried out following the protocol of Rapisarda et al. [27]. The procedure was carried out in a microplate reader (FLUOStar Omega, BMG Labtech, Ortenberg, Germany). A total of 20 μL of each sample were added in duplicate to each well of the 96-well plate and mixed with 280 μL of DPPH reagent (74 mg/L methanol prepared daily). The antioxidant reaction was monitored at 37 °C for 60 min and the calibration curve ranged from 0.01 to 0.4 mg of Trolox/mL. Results were expressed as mmol Trolox equivalent/kg food.

### 2.5. Statistical Analyses

Statistical differences were computed using unpaired Kruskal–Wallis test with a 95% confidence comparing the antioxidant capacity of each food group with the base mean antioxidant capacity (average antioxidant capacity presented by all groups). Therefore, we are showing whether one particular food group exhibits higher or lower antioxidant capacity than the average. A multivariate Principal Component Analysis (PCA) was carried out to explore differences between groups. Spearman parametric statistic was calculated to show the lineal relation between antioxidant capacity at a *p* value < 0.05. Statgraphics Plus software (Virginia, USA), version 5.1 was used to perform all the statistical analysis.

## 3. Results

Antioxidant capacity was measured in the supernatant obtained after in vitro digestion as well as in the one obtained after in vitro fermentation. Both of them account for the total antioxidant capacity of a given food. It is important to bear in mind that antioxidant values obtained after in vitro digestion are the same for both type of subjects, lean and obese, since food was only digested once and then fermented with both lean fecal material and obese fecal material. Overall, in vitro fermentation accounted for most of the total antioxidant capacity of the samples, as will be laid out in the following sections. Three different methods (Folin-Ciocalteu, FRAP and DPPH) were used to measure the antioxidant capacity. All values obtained were corrected for the antioxidant capacity provided by chemicals, enzymes and fecal material.

We checked for linear correlations using Pearson method between the different antioxidant assays. Considering all the values (n = 1472), we obtained significant (*p* < 0.05) correlations for the all the pairs: DPPH-FRAP (coefficient = 0.29), DPPH-FC (coefficient = 0.161) and FRAP-FC (coefficient = 0.632). 

### 3.1. Antioxidant Capacity Produced during In Vitro Digestion of Foods

**Figure 1:**
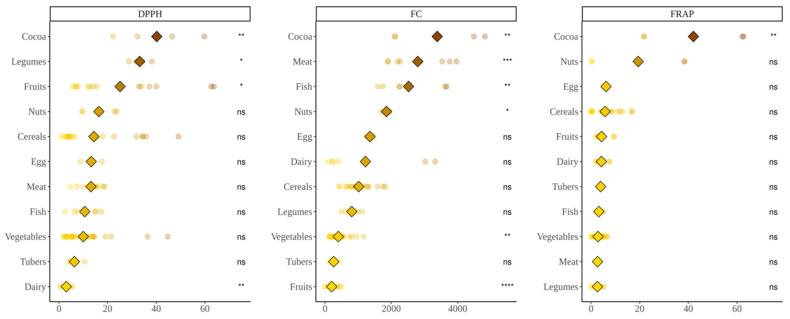
Antioxidant capacity of food obtained after in vitro digestion of selected foods. DPPH expressed as mmol Trolox equivalents/kg(L) of food; Folin–Ciocalteu (FC) expressed as mg equivalents of gallic acid/kg(L) of food; FRAP expressed as mmol Trolox equivalents/kg(L) of food. Diamonds represent the mean antioxidant capacity of each group. Dots represent the antioxidant capacity of each food within the group. Statistical analysis was performed via Kruskal–Wallis test. Each of the groups were compared to the average of all of them (i.e., base-mean). Statistic labels: *: *p* < 0.05, **: *p* < 0.01, ***: *p* < 0.001, ****: *p* < 0.0001, ns: not significant.

DPPH analysis (Figure 1) showed that cocoa products, legumes and fruits exerted significantly more antioxidant capacity than the average (*p* < 0.05) whereas dairy presented significantly lower values. The Folin–Ciocalteu assay (Figure 1) showed that cocoa products, fish, meat and nuts produced higher antioxidant capacity than the base-mean while fruits and vegetables were on the opposite side (*p* < 0.05). Regarding TEAC_FRAP_ (Figure 1), cocoa products again were the group with the highest antioxidant capacity. However, all the other food groups exhibited similar values with no significant differences versus the base-mean (*p* < 0.05).

As summary, in vitro digestion presented cocoa products as the food group with highest antioxidant potential in all three methods whereas opposite results were found for other food groups. There are two situations catching the eye: high Folin–Ciocalteu values achieved by meats and fish and low ones obtained by vegetables and fruits in some assays. This will be discussed in Section 4. 

### 3.2. Study of Antioxidant Capacity of Food Fermented with Fecal Material from Lean Children 

**Figure 2:**
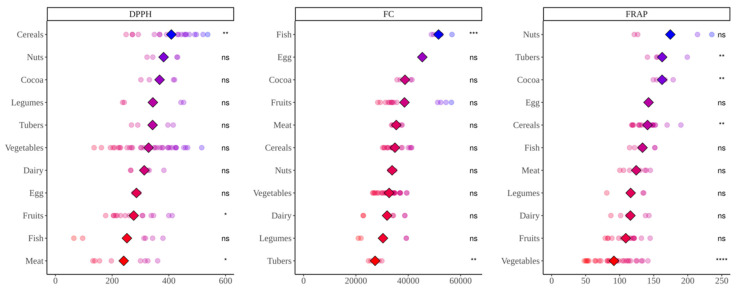
Antioxidant capacity of food obtained after in vitro fermentation of selected foods with fecal material from lean children. DPPH expressed as mmol Trolox equivalents/kg(L) of food; Folin–Ciocalteu (FC) expressed as mg equivalents of gallic acid/kg(L) of food; FRAP expressed as mmol Trolox equivalents/kg(L) of food. Diamonds represent the mean antioxidant capacity of each group. Dots represent the antioxidant capacity of each food within the group. Statistical analysis was performed via Kruskal–Wallis test. Each of the groups were compared to the average of all of them (i.e., base-mean). Statistic labels: *—*p* < 0.05, **—*p* < 0.01, ***—*p* < 0.001, ****—*p* < 0.0001, ns—not significant.

**Figure 3:**
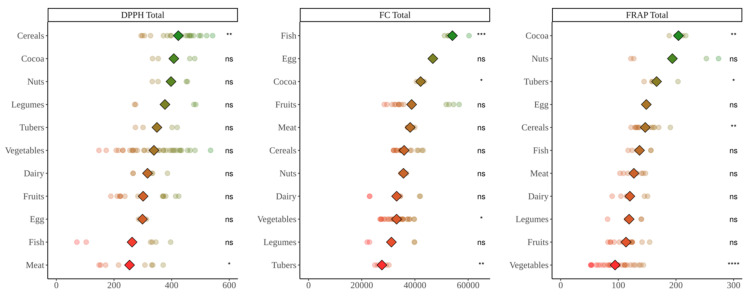
In vitro digestion values + in vitro fermentation values of food obtained after in vitro fermentation of selected foods with fecal material from lean children. DPPH expressed as mmol Trolox equivalents/kg(L) of food; Folin-Ciocalteu (FC) expressed as mg equivalents of gallic acid/kg(L) of food; FRAP expressed as mmol Trolox equivalents/kg(L) of food. Diamonds represent the mean antioxidant capacity of each group. Dots represent the antioxidant capacity of each food within the group. Statistical analysis was performed via Kruskal Wallis test. Each of the groups were compared to the average of all of them (i.e., base-mean). Statistic labels: *—*p* < 0.05, **—*p* < 0.01, ***—*p* < 0.001, ****—*p* < 0.0001, ns—not significant.

DPPH analysis (Figure 2) showed that cereals presented the highest antioxidant values (*p* < 0.05), while meat, fruits and fish (this one not significantly) exhibited the lowest values. When we consider total antioxidant capacity (Figure 3) (in vitro digestion values + in vitro fermentation values), results are very similar with cereals at the top and meat at the bottom. 

Folin–Ciocalteu assay (Figure 2) showed fish as the group with significantly higher antioxidant capacity compared to the average of all food groups (*p* < 0.05). Tubers, on the other hand, showed significantly lower values (*p* < 0.05). Here, again, it is surprising to have eggs or meats at similar level than fruits or cocoa, whereas legumes are among the lowest in antioxidant capacity. If we account for the antioxidant capacity released during digestion (Figure 3), fish is still the most antioxidant, though now cocoa products are also significantly above the base mean. Vegetables, however, join tubers with significantly lower values.

Regarding TEAC_FRAP_ (Figure 2), although nuts, as a group, showed the highest antioxidant capacity, only tubers and cocoa were significantly above the base-mean (*p* < 0.05), due to differences between different types of nuts. Vegetables showed, on the other hand, the lowest values. Similar results were obtained when considering total antioxidant capacity (Figure 3).

### 3.3. Study of Antioxidant Capacity of Food Fermented with Obese Fecal Material

**Figure 4:**
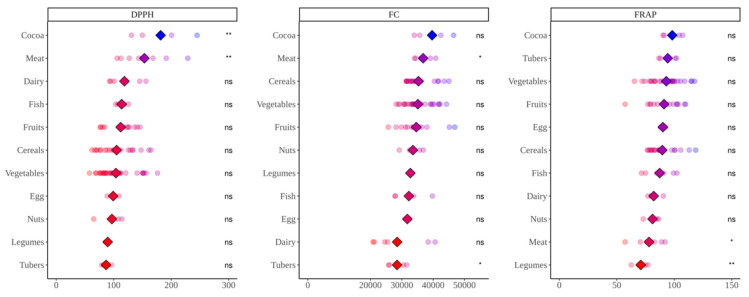
Antioxidant capacity of food obtained after in vitro fermentation of selected foods with fecal material from obese children. DPPH expressed as mmol Trolox equivalents/kg(L) of food; Folin–Ciocalteu (FC) expressed as mg equivalents of gallic acid/kg(L) of food; FRAP expressed as mmol Trolox equivalents/kg(L) of food. Diamonds represent the mean antioxidant capacity of each group. Dots represent the antioxidant capacity of each food within the group. Statistical analysis was performed via Kruskal–Wallis test. Each of the groups were compared to the average of all of them (i.e., base-mean). Statistic labels: *—*p* < 0.05, **—*p* < 0.01, ns—not significant.

**Figure 5:**
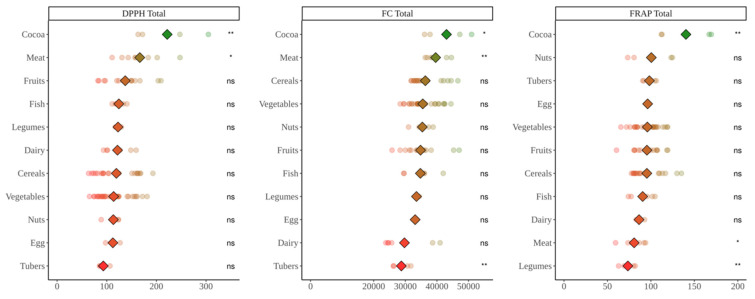
In vitro digestion values + in vitro fermentation values of food obtained after in vitro fermentation of selected foods with fecal material from obese children. DPPH expressed as mmol Trolox equivalents/kg(L) of food; Folin–Ciocalteu (FC) expressed as mg equivalents of gallic acid/kg(L) of food; FRAP expressed as mmol Trolox equivalents/kg(L) of food. Diamonds represent the mean antioxidant capacity of each group. Dots represent the antioxidant capacity of each food within the group. Statistical analysis was performed via Kruskal–Wallis test. Each of the groups were compared to the average of all of them (i.e., base-mean). Statistic labels: *—*p* < 0.05, **—*p* < 0.01, ns—not significant.

Regarding the DPPH assay (Figure 4), cocoa and meat showed the highest antioxidant capacity (*p* < 0.05). On the other hand, no food group showed significantly lower values than the average though tubers and legumes were the ones with lowest antioxidant capacity. Total antioxidant capacity showed similar results (Figure 5) though now eggs were second to the lowest. 

Regarding the Folin–Ciocalteu assay (Figure 4), meats showed the highest antioxidant values whereas tubers exhibited the lowest ones (*p* < 0.05). Cocoa products had a mean antioxidant capacity higher than meat products though probably due to differences between products, there was no significance. However, when considering total antioxidant capacity (Figure 5), cocoa was at the top with significantly higher values than the mean, followed by meats. 

For the FRAP assay (Figure 4), similar antioxidant capacity was achieved by all food groups with only meats and legumes showing values significantly lower than the base mean. However, after adding the antioxidant capacity released after in vitro digestion, cocoa showed the highest values with significant differences with the base mean whereas meat and legumes stayed at the bottom (Figure 5).

### 3.4. Comparison between Lean and Obese Subjects

**Figure 6:**
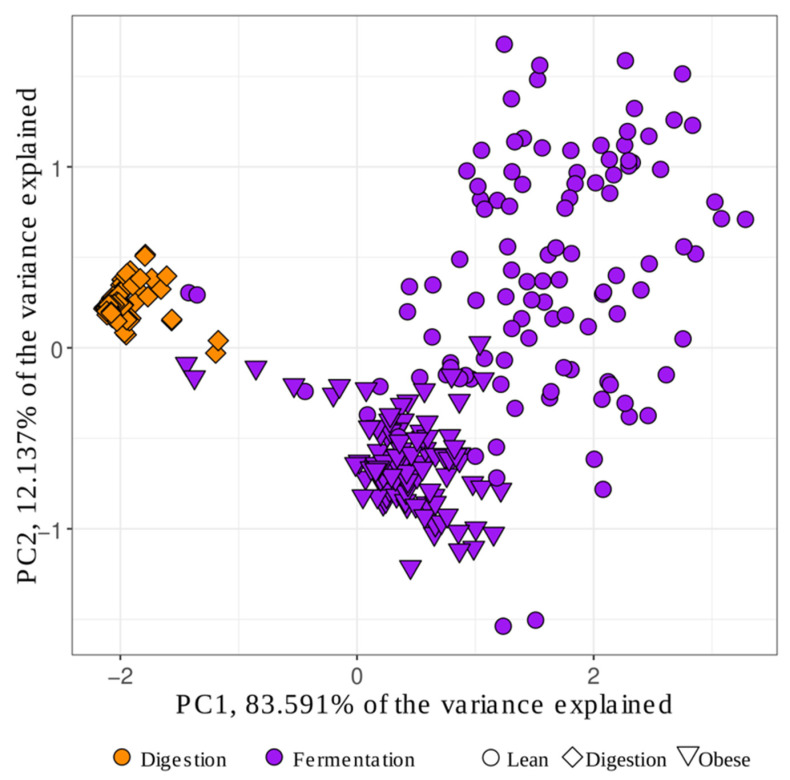
Principal Component Analysis of total antioxidant capacity obtained from different intervention groups with the three methods used (Folin–Ciocalteu, FRAP and DPPH).

A Principal Component Analysis (PCA) was carried out to study the distribution of the samples. Figure 6 shows the distribution of the samples in a two-dimensional plot. PCA represents, on one hand, that antioxidant capacity released during in vitro digestion was different than that released during in vitro fermentation (see the Section about contribution of each fraction to total antioxidant capacity). On the other hand, it also showed how samples belonging to each type of children mostly cluster together. PCA is therefore showing that the ability to release antioxidant capacity from foods could depend on the subject.

**Figure 7:**
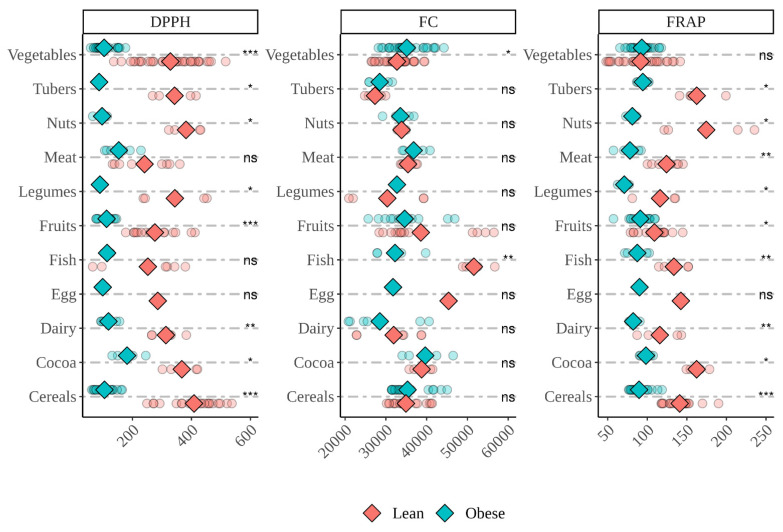
Comparison between the antioxidant capacity of different food groups after in vitro fermentation with fecal material from obese and lean children. DPPH expressed as mmol Trolox equivalents/kg(L) of food; Folin–Ciocalteu (FC) expressed as mg equivalents of gallic acid/kg(L) of food; FRAP expressed as mmol Trolox equivalents/kg(L) of food. Diamonds represent the mean antioxidant capacity of each group. Dots represent the antioxidant capacity of each food within the group. Statistical analysis was performed via Kruskal–Wallis test. Comparisons were made using “Lean” as the reference group. Statistic labels: *—*p* < 0.05, **—*p* < 0.01, ***—*p* < 0.001, ns—not significant.

Overall, when foods were fermented with fecal material from lean children, the antioxidant capacity released was higher, particularly in DPPH and FRAP assays (Figure 7). Folin–Ciocalteu assay presented very similar results for both type of subjects, and significant differences were only found in vegetables and fish. Fecal material from lean children yielded higher (*p* < 0.05) antioxidant capacity from vegetables (DDPH), tubers (DPPH and FRAP), nuts (DPPH and FRAP), meat (FRAP), legumes (DPPH and FRAP), fruits (DPPH and FRAP), fish (FC and FRAP), dairy products (DPPH and FRAP), cocoa products (DPPH and FRAP) and cereals (DPPH and FRAP). On the other hand, fecal material from obese children only exhibited higher (*p* < 0.05) antioxidant capacity when fermenting vegetables and measured via Folin–Ciocalteu. 

**Figure 8:**
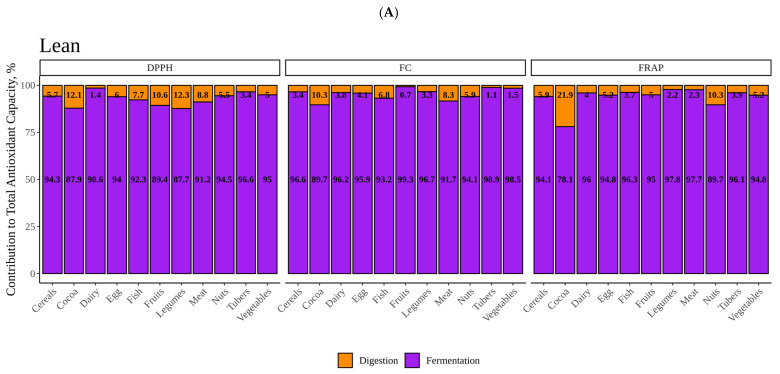

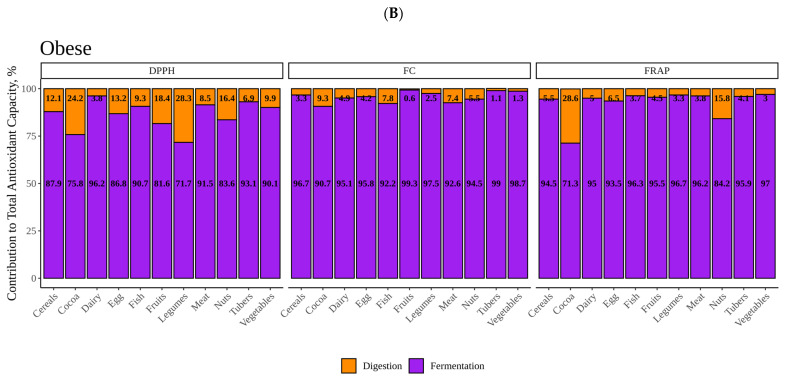
Contribution to the total antioxidant capacity of the fractions obtained after in vitro di- gestion and fermentation for lean and obese. (**A**) Contributions fermenting with fecal material from lean children. (**B**) Contributions fermenting with fecal material from obese children.

We also looked at how much each of the fractions (digestion and fermentation) contributed to total antioxidant capacity or, in other words, which process was able to extract the highest antioxidant power from the foods under study (Figure 8A,B). As we can see, for both, lean and obese, in vitro fermentation provided most of the antioxidant capacity. The contribution of digestion to total antioxidant capacity was higher in obese than in lean, especially when measured with DPPH, the reason being that obese fecal material generally yielded less antioxidant power.

## 4. Discussion

In this work, a total of 48 different foods of plant and animal origin were submitted to in vitro digestion followed by in vitro fermentation using fecal material from different donors: healthy children and obese children. Antioxidant capacity (DPPH, FRAP and Folin–Ciocalteu) was measured in the potentially absorbable fraction obtained after in vitro digestion as well as after gut microbial fermentation. 

Regarding in vitro digestion results, cocoa showed the highest antioxidant capacity for all three methodologies. Cocoa is known to possess a high amount of phytochemicals, especially phenolic compounds from the flavan-3-ol family such as catechin or epicatechin [28]. This could, in part, explain why cocoa show the highest antioxidant value as well as why fruits and legumes also showed significantly higher antioxidant capacity than the base-mean with DPPH. Vegetables, on the other hand, was surprisingly found as one of the least antioxidant group of foods. It has been found that due to the nature of vegetables’ cell wall, these are hard to break down during digestion which could lead to a reduced release of its content and, thus, phytochemicals during gastrointestinal digestion [29]. Some more aggressive cooking methods such as grilling or frying would help making the structure softer so it can more easily be broken down and release more antioxidant capacity during digestion [29]. Similar behavior for vegetables was found across all three methods.

However, meats and fish, followed by nuts, also exhibited significantly higher antioxidant capacity than the base-mean when antioxidant capacity was measured via FC. At the same time, vegetables, legumes and fruits showed lower values than the base-mean, only significant for vegetables and fruits. These results did not fall within expectations. The Folin–Ciocalteu assay is often used to estimate total polyphenol content [25]. However, it has been found that reducing compounds interact with the reagent regardless of their actual antioxidant power. Among those compounds we can find sugars or amino acids [28]. Therefore, the actual phenolic content could be overestimated [30]. In order to test this possibility, we perform a correlation test between FC values a protein content for all 48 foods, finding it to be large (r = 0.8339) and significant (*p* < 0.05).

FRAP, on the other hand, did not show much variation between different food categories with the exception of cocoa and nuts, though the latter did not show significantly higher antioxidant values than the rest. These results would make sense regarding animal products which are not known for their antioxidant potential and would also agree with DPPH.

Regarding in vitro fermentation with fecal material from lean children, cocoa was once again among the top three foods with the highest antioxidant capacity, though it was only significantly in the case of FRAP. Nuts were also among those top three when using DPPH and FRAP methods, though only significantly for DPPH. As commented above, cocoa [28] as well as nuts are known carriers of phytochemicals such as phenolics [31]. High-protein foods were among the ones with the lowest antioxidant values regarding DPPH, whereas vegetables were situated half way, probably due to large differences between specific vegetable foods. Fruits also showed significantly lower antioxidant potential than the base mean although here too there were large differences within the group. Cereals were the food group that showed to be significantly above the base mean for DPPH method. Comparatively speaking, vegetables are now, after fermentation, producing more antioxidant capacity than during digestion. This could indicate that their microbial degradation could help releasing antioxidants that otherwise would be inaccessible. 

FC showed similar results to those obtained during in vitro digestion with high-protein foods showing some of the highest antioxidant values, though only fish was statistically significant (*p* < 0.05). Here, again, we found a statistically significant correlation between protein content and FC values (r = 0.3942; *p* < 0.05). However, here is not clear whether high antioxidant values are due to interactions as described above or to actual antioxidant compounds. It has been proven before that several bacterial species such as different *Bacteroides, Eubacterium hallii* or *Clostridium barlettii* can metabolize aromatic amino acids into small phenolic compounds, same as the ones produced in plants [32,33]. These include phenylpropionic acids, phenylacetic acids or 4-hydroxy-phenylacetic acid [32,33]. Therefore, though FC values could indeed be overestimated, some of the antioxidant capacity registered could come from those phenolics. However, low DPPH and FRAP values of animal foods could indicate that in this case, FC is actually overestimating antioxidant power.

As stated above, cocoa was, along with tubers and nuts (this one not significantly) found to have a higher antioxidant power when measured via FRAP. The rest of the groups showed similar antioxidant capacity and only vegetables were significantly below the base mean. Although there are great differences within the group, some vegetables did show low antioxidant values whereas others displayed values comparable to other groups. 

**Figure 9:**
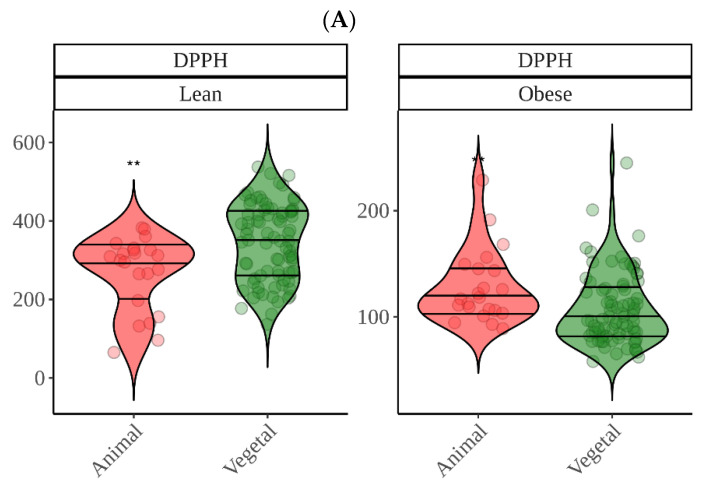

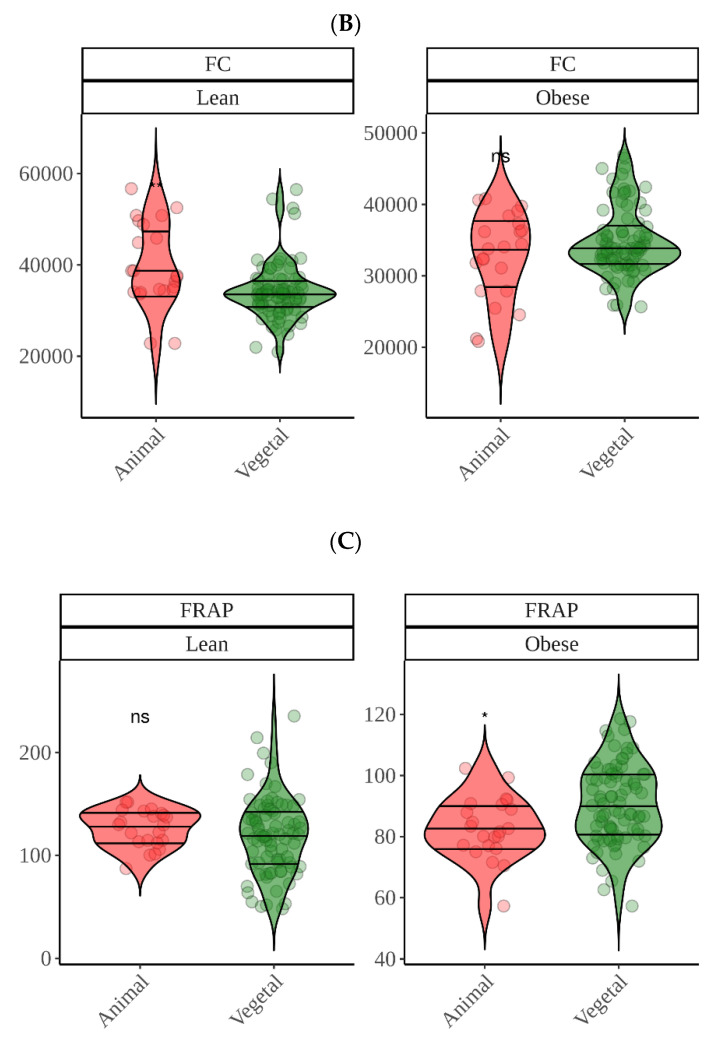
Antioxidant capacity released by lean or obese gut microbiota from plant-origin foods and animal products. (**A**) DPPH; (**B**) Folin–Ciocalteu; (**C**) FRAP. Statistical analysis was performed via Kruskal Wallis test. Comparisons were made using “Vegetal” as the reference group. Solid horizontal lines within shapes show 0.25, 0.5, and 0.75 quartiles. Statistic labels: *—*p* < 0.05, **—*p* < 0.01, ns—not significant.

Regarding in vitro fermentation with fecal material from obese children, cocoa was once more the most antioxidant group though only significantly in the case of DPPH. We can probably suggest now that, overall, cocoa was the most antioxidant food. DPPH showed that high protein foods were among the most antioxidant food groups, which was, in part, opposite to what we found using lean fecal material. Since the DPPH reagent has not been reported to react with proteins (as FC), we tried to find another explanation. This finding could be related to differences between gut microbiota from lean and obese. As depicted in Figure 5, whereas meat is second to cocoa using obese fecal material, when we use lean gut microbiota the result is opposite and is, in fact, where differences between lean and obese are the lowest (Figure 5). Actually, while lean gut microbiota can extract significantly more antioxidant capacity from plant-origin foods, obese gut microbiota does the opposite and extracts significantly more from animal products (Figure 9). There have been several reports that have consistently found specific features of obese gut microbiota, probably the most apparent is the depletion in *Bacteroidetes* and higher abundance of *Firmicutes* [34,35]. This would lead to a reduced ability to ferment plant polysaccharides when compared to a lean microbiota [36]. Therefore, breakdown of vegetable cells would become harder and lower concentrations of phytochemicals would be released. Obesity has also been associated to high protein, high fat and low fiber diets which could suggests that proteolytic pathways in gut microbes are favored [37]. However, this would only explain why lean gut microbiota extract more antioxidant capacity from plant origin foods. Riadaura et al. [36] carried out an experiment that involved a fecal transplantation to gnobiotic mice from twins discordant for obesity. These authors found higher concentrations of branched chain amino acids and some others including phenyalanine and tyrosine in plasma, which could indicate lower degradation and formation of small phenolics. Then again, it has also been consistently found that obese gut microbiota has an increased production of phenilalanine, tyrosine and tryptophan [34,35]. Therefore, whether these amino acids are coming from food protein degradation or not requires further study. 

However, when antioxidant capacity was measured via FRAP and FC using obese gut microbiota, though meat showed high values via FC, animal foods presented in general lower values than plant-origin products. Nevertheless, FC values were similar among samples and only tubers and meats were significantly different to the base mean. When comparing FC values between lean and obese samples, they were very similar, only showing differences for egg and fish, which would require further and deeper research. FRAP, on the other hand, exhibited higher antioxidant values when fermenting with lean fecal material except for vegetables, though fruit values were also close to one another. Although this coincides with DPPH, the specific behavior of the different food groups was different than that found for DPPH. FRAP showed that lean gut microbiota produced similar antioxidant capacity from animal and plant origin products whereas obese gut microbiota produced higher values from plant products. Whether this behavior is gut microbe-related or due to the assays’ chemistry is hard to conclude and additional experiments with more subjects from each group would be needed. However, regarding the antioxidant assays chemistry, pH could be playing an important role here. Whereas DPPH is carried out at pH 7, FRAP is carried out at pH 3.6. Both assays are based on the same redox principle, transference of electrons from the antioxidant to the radical (DPPH) or to the metal (FRAP) [38]. However, polyphenols should be more antioxidant at pH closer to 7 because deprotonation of phenols increase with pH and, therefore, electron transfer is easier [39]. Accordingly, lean gut microbiota could be releasing more phenolics from vegetables or fruits but they would not be detected, resulting in similar values to those obtained with obese children. That could also be true for small phenolics obtained from amino acid degradation. Regardless, whether this is the reason or it lies in the metabolism of gut microbes would, as commented above, require further investigation with a larger cohort. 

## 5. Conclusions

In conclusion, we evaluated a number of foods grouped in several categories. Cocoa products (dark chocolate and cocoa butter) displayed, overall, the highest antioxidant capacity. Antioxidant capacity of foods is released in a two-step process: first, some of this antioxidant capacity is released during digestion, and, usually, more antioxidant power released from those foods more easily digested whereas others, such as vegetables, release lower concentrations. Regardless, most of the antioxidant capacity is released during gut microbial fermentation, which could be due to two main reasons: for plant-origin foods (hard to digest due to lack of enzymes) most phytochemicals could be released only after gut microbes have broken down vegetal cells. Secondly, animal products that lack phytochemicals (they could have some due to animal feed) could exhibit their antioxidant power after amino acids are fermented into small phenolics but not before as larger peptides. Additionally, large differences were detected depending on whether foods were fermented with lean or obese fecal material. Overall, when using lean gut microbiota, antioxidant capacity released was higher, though this was only true for DPPH and FRAP methods. Moreover, according to DPPH results, lean gut microbiota could potentially release more antioxidant power from vegetables than from animal products, while obese gut microbiota did the opposite. FRAP, on the other hand, showed how it was obese gut microbiota the one that released higher levels of antioxidant power from plants products. Although this could be related to the chemistry of the antioxidant assay, it is not clear and further investigation would help elucidate it. Regardless, different antioxidant assays could show different antioxidant behaviors although FC has showed a potential interaction with proteins or amino acids which would limit its usefulness. 

## Data Availability

Not applicable.
